# 1,2,3,4,5,6-Hexa-*O*-acetyl-*scyllo*-inositol

**DOI:** 10.1107/S1600536812033600

**Published:** 2012-07-28

**Authors:** Rajni Kant, Vivek K. Gupta, Kamini Kapoor, Renu Chib, Bhahwal A. Shah, Subhash C. Taneja

**Affiliations:** a X-ray Crystallography Laboratory, Post-Graduate Department of Physics & Electronics, University of Jammu, Jammu Tawi 180 006, India; b Natural Product Microbes Division, Indian, Institute of Integrative Medicine, Canal Road, Jammu Tawi 180 001, India

## Abstract

The title mol­ecule, C_18_H_24_O_12_, has crystallographic 2/*m* symmetry with two acetate group located on a mirror plane. The H—C*sp* ^3^—O—C*sp* ^2^ torsion angles characterizing orientation of the acetyl groups with respect to the cyclo­hexane ring are 0.0, 23.9 and −23.9°. The cyclo­hexane ring is in a chair conformation with all substituents in equatorial positions. In the crystal, mol­ecules are connected through C—H⋯O hydrogen bonds into a chain extending along the *c* axis.

## Related literature

For applications of the title compound, see: Kamal & Mathur (1991 ▶); Anonymous *et al.* (2001 ▶, 2003 ▶). For related structures, see: Abboud *et al.* (1990 ▶).
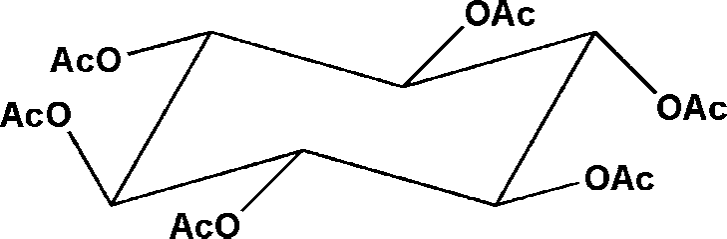


## Experimental

### 

#### Crystal data

C_18_H_24_O_12_
*M* *_r_* = 432.37
Monoclinic, 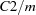

*a* = 12.901 (3) Å
*b* = 14.013 (3) Å
*c* = 5.8572 (12) Å
β = 97.33 (2)°
*V* = 1050.2 (4) Å^3^
*Z* = 2
Mo *K*α radiation
μ = 0.12 mm^−1^
*T* = 293 K
0.3 × 0.2 × 0.2 mm

#### Data collection

Oxford Diffraction Xcalibur Sapphire3 diffractometer
Absorption correction: multi-scan (*CrysAlis PRO*; Oxford Diffraction, 2010 ▶) *T*_min_ = 0.472, *T*_max_ = 1.000
3652 measured reflections
959 independent reflections
626 reflections with *I* > 2σ(*I*)
*R*_int_ = 0.081

#### Refinement

*R*[*F* ^2^ > 2σ(*F* ^2^)] = 0.061
*wR*(*F* ^2^) = 0.198
*S* = 1.08
959 reflections
77 parameters
H-atom parameters constrained
Δρ_max_ = 0.37 e Å^−3^
Δρ_min_ = −0.30 e Å^−3^

### 

Data collection: *CrysAlis PRO* (Oxford Diffraction, 2010 ▶); cell refinement: *CrysAlis PRO*; data reduction: *CrysAlis PRO*; program(s) used to solve structure: *SHELXS97* (Sheldrick, 2008 ▶); program(s) used to refine structure: *SHELXL97* (Sheldrick, 2008 ▶); molecular graphics: *ORTEP-3* (Farrugia, 1997 ▶); software used to prepare material for publication: *PLATON* (Spek, 2009 ▶).

## Supplementary Material

Crystal structure: contains datablock(s) I, global. DOI: 10.1107/S1600536812033600/gk2511sup1.cif

Structure factors: contains datablock(s) I. DOI: 10.1107/S1600536812033600/gk2511Isup2.hkl

Additional supplementary materials:  crystallographic information; 3D view; checkCIF report

## Figures and Tables

**Table 1:** Hydrogen-bond geometry (Å, °)

*D*—H⋯*A*	*D*—H	H⋯*A*	*D*⋯*A*	*D*—H⋯*A*
C1—H1⋯O4^i^	0.98	2.57	3.393 (4)	141

Symmetry code: (i) 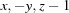.

**Fig. 1.**
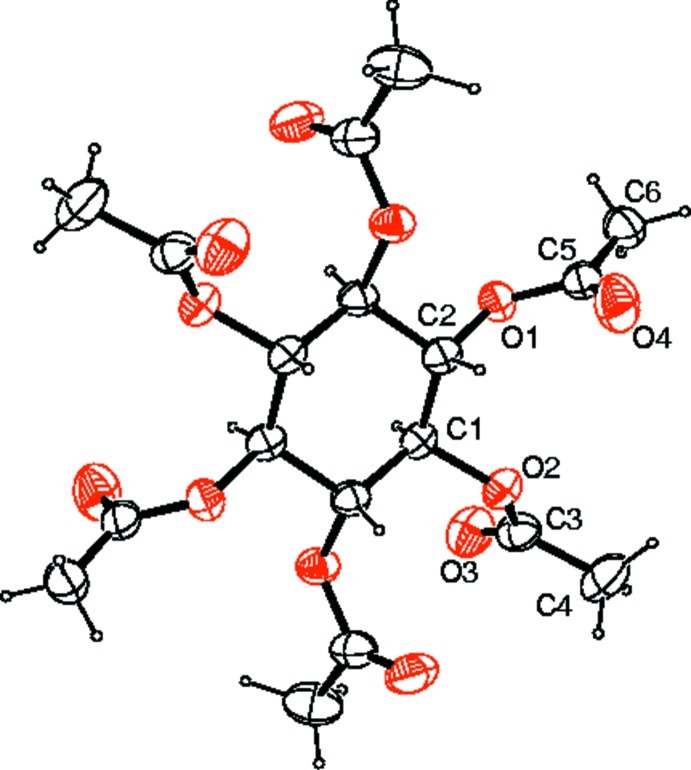
ORTEP view of the molecule with the atom-labeling scheme. The displacement ellipsoids are drawn at the 40% probability level. H atoms are shown as small spheres of arbitrary radii. Symmetry operations generating the whole molecule from the asymmetric part are: 1-x, -y, 1-z and x, -y, z.

**Fig. 2.**
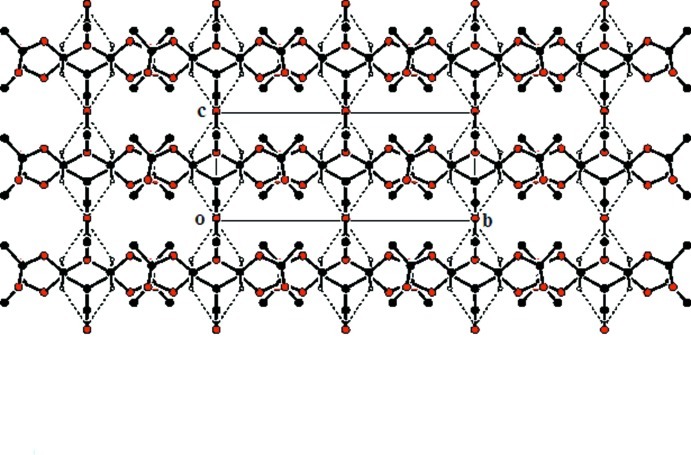
The packing arrangement of molecules viewed down the a axis. For clarity, hydrogen atoms which are not involved in hydrogen bonding have been omitted.

## supplementary crystallographic information

### Comment

Parthenium hysterophorus (Asteraceae) is an annual weed. It is commonly known as congress weed, carrot weed, star weed, feverfew *etc*. A decoction of the root of P. hysterophorus finds use in treatment of dysentery (Anonymous *et al.*, 2001). Histamine (0.35%) is present in the roots of plant (Kamal & Mathur, 1991). The roots contain parthenin, caffeic, chlorogenic, *p*-hydroxybenzoic, *p*-anisic, vanilic, salicylic, gentisic, neo-chlorogenic and proto-catechuic acids (Anonymous *et al.*, 2003). Roots of this plant were not that much explored thus making this an interesting field to carry out further studies.

In the title compound (Fig. 1), all bond lengths and angles are normal and correspond to those observed in related structure (Abboud *et al.*, 1990). The twist angles characterizing orientation of the acetyl group with respect to the cyclohexane ring are: H1—C1—O2—C3 = -23.9°, H2—C2—O1—C5 = 0.0°. The cyclohexyl ring is in the chair conformation. Intermolecular C—H···O hydrogen bonds (Table 1) link the molecules into chains along the c axis..

### Experimental

Dried roots (2 kg) of P. hysterophorus was crushed, water extract was prepared and lyophilized. Dry powder (5 g) obtained was treated with pyridine (50 ml) and acetic anhydride (60 ml) and was stirred at room temperature. Progress of the reaction was checked by TLC. After completion of the reaction, mixture was treated dropwise with 1.5 N aqueous HCl (50 ml) and then extracted with ethyl acetate (3x100mL). After usual workup and removal of the solvents the reaction product obtained was 8 g. Chromatographic separation of the products on a silica gel column using chloroform: methanol (99:1 to 80:20) as eluent, gave pure myo-inositol hexaacetate and later fractions gave scyllo-inositol hexaacetate as white crystals m.p. 651–653 K. Single crystal of the compound was grown by slow evaporation technique using methanol /chloroform as the solvent system.

### Refinement

All H atoms (except methyl C6 H) were positioned geometrically and were treated as riding on their parent C atoms, with C—H distances of 0.96–0.98 Å and with *U*_iso_(H) = 1.2*U*_eq_(C) or 1.5*U*_eq_(methyl C).

### Crystal data

**Table:**

C_18_H_24_O_12_	*F*(000) = 456
*M**_r_* = 432.37	*D*_x_ = 1.367 Mg m^−^^3^
Monoclinic, *C*2/*m*	Mo *K*α radiation, λ = 0.71073 Å
Hall symbol: -C 2y	Cell parameters from 1553 reflections
*a* = 12.901 (3) Å	θ = 3.5–29.1°
*b* = 14.013 (3) Å	µ = 0.12 mm^−^^1^
*c* = 5.8572 (12) Å	*T* = 293 K
β = 97.33 (2)°	Block, white
*V* = 1050.2 (4) Å^3^	0.3 × 0.2 × 0.2 mm
*Z* = 2	

### Data collection

**Table:**

Oxford Diffraction Xcalibur Sapphire3 diffractometer	959 independent reflections
Radiation source: fine-focus sealed tube	626 reflections with *I* > 2σ(*I*)
Graphite monochromator	*R*_int_ = 0.081
Detector resolution: 16.1049 pixels mm^-1^	θ_max_ = 25.0°, θ_min_ = 3.5°
ω scan	*h* = −15→15
Absorption correction: multi-scan (*CrysAlis PRO*; Oxford Diffraction, 2010)	*k* = −16→16
*T*_min_ = 0.472, *T*_max_ = 1.000	*l* = −6→6
3652 measured reflections	

### Refinement

**Table:**

Refinement on *F*^2^	Primary atom site location: structure-invariant direct methods
Least-squares matrix: full	Secondary atom site location: difference Fourier map
*R*[*F*^2^ > 2σ(*F*^2^)] = 0.061	Hydrogen site location: inferred from neighbouring sites
*wR*(*F*^2^) = 0.198	H-atom parameters constrained
*S* = 1.08	*w* = 1/[σ^2^(*F*_o_^2^) + (0.1032*P*)^2^*P*] where *P* = (*F*_o_^2^ + 2*F*_c_^2^)/3
959 reflections	(Δ/σ)_max_ = 0.001
77 parameters	Δρ_max_ = 0.37 e Å^−^^3^
0 restraints	Δρ_min_ = −0.30 e Å^−^^3^

### Special details

**Table:**

Experimental. Absorption correction: *CrysAlis PRO*, Oxford Diffraction Ltd., Version 1.171.34.40 (release 27–08-2010 CrysAlis171. NET) (compiled Aug 27 2010,11:50:40) Empirical absorption correction using spherical harmonics, implemented in SCALE3 ABSPACK scaling algorithm.
Geometry. All e.s.d.'s (except the e.s.d. in the dihedral angle between two l.s. planes) are estimated using the full covariance matrix. The cell e.s.d.'s are taken into account individually in the estimation of e.s.d.'s in distances, angles and torsion angles; correlations between e.s.d.'s in cell parameters are only used when they are defined by crystal symmetry. An approximate (isotropic) treatment of cell e.s.d.'s is used for estimating e.s.d.'s involving l.s. planes.
Refinement. Refinement of *F*^2^ against ALL reflections. The weighted *R*-factor *wR* and goodness of fit *S* are based on *F*^2^, conventional *R*-factors *R* are based on *F*, with *F* set to zero for negative *F*^2^. The threshold expression of *F*^2^ > σ(*F*^2^) is used only for calculating *R*-factors(gt) *etc*. and is not relevant to the choice of reflections for refinement. *R*-factors based on *F*^2^ are statistically about twice as large as those based on *F*, and *R*- factors based on ALL data will be even larger.

### Fractional atomic coordinates and isotropic or equivalent isotropic displacement parameters (Å^2^)

**Table:**

	*x*	*y*	*z*	*U*_iso_*/*U*_eq_	Occ. (<1)
O1	0.3027 (2)	0.0000	0.6341 (4)	0.0412 (8)	
O2	0.42280 (15)	0.16670 (15)	0.6695 (3)	0.0448 (7)	
O3	0.3640 (2)	0.26466 (17)	0.3790 (4)	0.0660 (8)	
O4	0.3169 (3)	0.0000	1.0179 (5)	0.0722 (12)	
C1	0.4426 (2)	0.0905 (2)	0.5150 (4)	0.0373 (8)	
H1	0.3991	0.0978	0.3662	0.045*	
C2	0.4142 (3)	0.0000	0.6355 (6)	0.0376 (10)	
H2	0.4485	0.0000	0.7947	0.045*	
C3	0.3838 (2)	0.2500 (2)	0.5799 (6)	0.0483 (9)	
C4	0.3714 (3)	0.3192 (3)	0.7664 (6)	0.0695 (12)	
H4A	0.4360	0.3526	0.8080	0.104*	
H4B	0.3527	0.2857	0.8981	0.104*	
H4C	0.3174	0.3641	0.7136	0.104*	
C5	0.2635 (4)	0.0000	0.8365 (8)	0.0450 (11)	
C6	0.1486 (4)	0.0000	0.7967 (8)	0.0566 (13)	
H6A	0.1257	0.0000	0.6342	0.085*	
H6B	0.1226	0.0559	0.8650	0.085*	0.50
H6C	0.1226	−0.0559	0.8650	0.085*	0.50

### Atomic displacement parameters (Å^2^)

**Table:**

	*U*^11^	*U*^22^	*U*^33^	*U*^12^	*U*^13^	*U*^23^
O1	0.0386 (16)	0.0466 (18)	0.0389 (16)	0.000	0.0071 (12)	0.000
O2	0.0514 (14)	0.0377 (13)	0.0449 (12)	0.0038 (10)	0.0042 (10)	−0.0055 (9)
O3	0.0836 (19)	0.0463 (15)	0.0667 (17)	0.0192 (14)	0.0036 (13)	0.0052 (12)
O4	0.078 (3)	0.095 (3)	0.045 (2)	0.000	0.0124 (17)	0.000
C1	0.0416 (17)	0.0317 (16)	0.0377 (16)	0.0024 (14)	0.0020 (13)	−0.0030 (12)
C2	0.040 (2)	0.034 (2)	0.038 (2)	0.000	0.0020 (16)	0.000
C3	0.0431 (18)	0.0376 (19)	0.063 (2)	0.0026 (15)	0.0026 (15)	−0.0027 (15)
C4	0.071 (2)	0.050 (2)	0.086 (3)	0.006 (2)	0.001 (2)	−0.0216 (19)
C5	0.057 (3)	0.031 (2)	0.049 (3)	0.000	0.016 (2)	0.000
C6	0.056 (3)	0.044 (3)	0.076 (3)	0.000	0.029 (2)	0.000

### Geometric parameters (Å, º)

**Table:**

O1—C5	1.347 (5)	C2—H2	0.9800
O1—C2	1.438 (5)	C3—C4	1.485 (4)
O2—C3	1.350 (4)	C4—H4A	0.9600
O2—C1	1.443 (3)	C4—H4B	0.9600
O3—C3	1.189 (4)	C4—H4C	0.9600
O4—C5	1.190 (5)	C5—C6	1.471 (6)
C1—C1^i^	1.514 (5)	C6—H6A	0.9600
C1—C2	1.519 (3)	C6—H6B	0.9600
C1—H1	0.9800	C6—H6C	0.9600
C2—C1^ii^	1.519 (3)		
			
C5—O1—C2	118.8 (3)	O2—C3—C4	110.4 (3)
C3—O2—C1	118.8 (2)	C3—C4—H4A	109.5
O2—C1—C1^i^	109.09 (19)	C3—C4—H4B	109.5
O2—C1—C2	104.7 (2)	H4A—C4—H4B	109.5
C1^i^—C1—C2	110.6 (2)	C3—C4—H4C	109.5
O2—C1—H1	110.8	H4A—C4—H4C	109.5
C1^i^—C1—H1	110.8	H4B—C4—H4C	109.5
C2—C1—H1	110.8	O4—C5—O1	123.1 (4)
O1—C2—C1^ii^	107.3 (2)	O4—C5—C6	126.7 (4)
O1—C2—C1	107.3 (2)	O1—C5—C6	110.1 (4)
C1^ii^—C2—C1	113.3 (3)	C5—C6—H6A	109.5
O1—C2—H2	109.6	C5—C6—H6B	109.5
C1^ii^—C2—H2	109.6	H6A—C6—H6B	109.5
C1—C2—H2	109.6	C5—C6—H6C	109.5
O3—C3—O2	123.8 (3)	H6A—C6—H6C	109.5
O3—C3—C4	125.8 (3)	H6B—C6—H6C	109.5
			
C3—O2—C1—C1^i^	98.2 (3)	O2—C1—C2—C1^ii^	−172.6 (2)
C3—O2—C1—C2	−143.4 (3)	C1^i^—C1—C2—C1^ii^	−55.2 (4)
C5—O1—C2—C1^ii^	119.0 (2)	C1—O2—C3—O3	0.4 (5)
C5—O1—C2—C1	−119.0 (2)	C1—O2—C3—C4	−178.8 (2)
O2—C1—C2—O1	69.1 (3)	C2—O1—C5—O4	0.0
C1^i^—C1—C2—O1	−173.5 (2)	C2—O1—C5—C6	180.0

Symmetry codes: (i) −*x*+1, *y*, −*z*+1; (ii) *x*, −*y*, *z*.

### Hydrogen-bond geometry (Å, º)

**Table:**

*D*—H···*A*	*D*—H	H···*A*	*D*···*A*	*D*—H···*A*
C1—H1···O4^iii^	0.98	2.57	3.393 (4)	141

Symmetry code: (iii) *x*, −*y*, *z*−1.
